# Irisin enhances chondrogenic differentiation of human mesenchymal stem cells via Rap1/PI3K/AKT axis

**DOI:** 10.1186/s13287-022-03092-8

**Published:** 2022-08-03

**Authors:** Taiqiu Chen, Yan Peng, Wenjun Hu, Huihong Shi, Pengfei Li, Yichen Que, Jincheng Qiu, Xianjian Qiu, Bo Gao, Hang Zhou, Yanbo Chen, Yuanxin Zhu, Shaoguang Li, Anjing Liang, Wenjie Gao, Dongsheng Huang

**Affiliations:** Department of Orthopedics, Sun Yat-Sen Memorial Hospital of Sun Yat-Sen University, #107 West Yan Jiang Road, Guangzhou, Guangdong China

**Keywords:** Irisin, Human mesenchymal stem cells, Chondrogenic differentiation, Rap1/PI3K/AKT signaling pathway, *SIPA1L2*, *miR-125b-5p*

## Abstract

**Background:**

Human mesenchymal stem cells (hMSCs) have been proven to have inherent chondrogenic differentiation potential, which appears to be used in cartilage regeneration. Increasing evidence suggests that irisin enhances osteoblast differentiation of MSCs, but little is known about its potential on chondrogenic differentiation.

**Methods:**

In the study, we investigated the effects of irisin on chondrogenic differentiation of hMSCs using a high-density pellet culture system. The cartilage pellets were evaluated by morphology, and the metabolism of cartilage matrix was detected by qPCR, western blot and immunohistochemistry. Next, RNA-seq was performed to explore the underlying mechanism. Furthermore, using the transduction of plasmid, miRNAs mimics and inhibitor, the activation of Rap1/PI3K/AKT axis, the expression level of SIPA1L2, and the functional verification of *miR-125b-5p* were detected on day 7 of chondrogenic differentiation of hMSCs.

**Results:**

Compared with the controls, we found that irisin treatment could significantly enhance the chondrogenic differentiation of hMSCs, enlarge the induced-cartilage tissue and up-regulate the expression levels of cartilage markers. RNA-seq indicated that irisin activated the Rap1 and PI3K/AKT signaling pathway, and the lower expression level of SIPA1L2 and the higher expression level of *miR-125b-5p* were found in irisin-treated group. Further, we found that irisin treatment could up-regulate the expression level of *miR-125b-5p*, targeting *SIPA1L2* and consequently activating the Rap1/PI3K/AKT axis on the process of chondrogenic differentiation of hMSCs.

**Conclusions:**

Collectively, our study reveals that irisin can enhance chondrogenic differentiation of hMSCs via the Rap1/PI3K/AKT pathway, suggesting that irisin possesses prospects in cartilage regeneration.

**Supplementary Information:**

The online version contains supplementary material available at 10.1186/s13287-022-03092-8.

## Introduction

Cartilage damage is very commonly encountered in patients with poor self-healing due to the lack of blood vessels, lymph, and nerves [1, 2]. Many diseases including osteoarthritis, burns, and congenital deformities are characterized by cartilage defects, which lead to dysfunction and even disability [3, 4]. Autologous cartilage transplantation has been a valid technique in repairing injured cartilage, which faces restricted proliferative capacity [5, 6]. Thus, searching for suitable and effective methods to maintain the chondrocyte phenotype is essential for cartilage repair. Human mesenchymal stem cells (hMSCs) have been proven to be an effective cartilage tissue repair reagent due to their capacities of intrinsic regeneration and chondrogenic differentiation [7, 8].

Irisin is a polypeptide fragment formed by cleavage of type III fibronectin component including protein 5 (FNDC5) as the extracellular receptor ectodomain, which is widely secreted by multiple tissues such as skeletal muscle, liver, salivary glands, cardiomyocytes, and bone [9–11]. Accumulated studies have revealed that irisin was involved in many physiological processes, such as lipid and glucose homeostasis, anti-oxidation, and anti-tumor activities [12–15]. Regarding the regulation of development and metabolism of the skeletal system, irisin was discovered to possess a powerful regulative effect. In vivo, irisin treatment prevented loss of bone mass and improved bone metabolism [16, 17], while in vitro, irisin has been found to enhance the osteoblastogenetic process of MSCs, respectively [18, 19]. In addition, Li et al. [20] discovered that irisin was involved in the development of articular cartilage, and Wang et al. [21] found that irisin displayed chondroprotective effects on the development of osteoarthritis. Despite its considerable roles in the regulation of skeletal metabolism, it remains elusive whether irisin regulated the chondrogenic differentiation of hMSCs.

In the study, the ability to enhance chondrogenic differentiation of hMSCs of irisin and its potential underlying mechanism are evaluated. Herein, we report that irisin enhances chondrogenic differentiation of hMSCs by up-regulating the expression level of *miR-125b-5p,* targeting *SIPA1L2,* and activating Rap1/PI3K/AKT signaling. Our results provide abundant evidence for the use of irisin as a repair reagent on diseases with cartilage injury.

## Material and methods

### Isolation and culture of human mesenchymal stem cells

As previously described, hMSCs were obtained from healthy volunteer donors, and isolated from bone marrow [22–24]. The bone marrow samples were diluted with PBS, and the cells were then fractionated. Then, the cells were resuspended in low-glucose DMEM containing 10% fetal serum, seeded, and incubated. The non-adherent cells were removed after 48 h. When they reached approximately 80% confluence, the cells were trypsinized, and plated again for the following experiments. Our research was approved by the Institutional Research Ethical Committee of Sun Yat-sen University (Approval number: SYSEC-KY-KS-2021-065).

### Chondrogenic differentiation

As previously described [22–24], briefly, hMSCs were trypsinized, washed, and then resuspended at a density of about 2 × 10^7^ cells/mL in 24 wells. They were cultured with human mesenchymal stem cell chondrogenic differentiation medium containing dexamethasone, ascorbate, ITS + supplement, sodium pyruvate, proline, and TGF-β3 (Cyagen Biosciences Inc.). When the cells adhered, 500 μL medium with or without r-irisin (100 ng/ml) was added. The medium was changed every 3 days, and the induced-cartilage tissues were collected on day 7 and 14.

### Transcriptome sequencing and bioinformatics analysis

To identify the differential expression of RNA transcripts between controls and irisin-treated groups, genome‐wide transcriptional sequencing was performed by Epibiotek (Guangzhou Co.,Ltd.). Differential expression analysis between controls and irisin-treated groups was performed using the DESeq2 R package. *P* value was adjusted using the Hochberg and Benjamini’s approach in order to control the false discovery rate. The gene expression levels were regarded to be statistically significant when the adjusted *P* value < 0.05 found by DESeq2. Next, Kyoto Encyclopedia of Genes and Genomes (KEGG) pathways enrichment analysis and Gene Ontology (GO) enrichment analysis were performed on the website (https://david.ncifcrf.gov). The raw data of transcriptome sequencing had been submitted to the GEO (GSE201594).

### Reverse transcription and real-time PCR

Through the high-throughput tissue grinder, total RNAs were extracted from cartilage tissues. According to the manufacturer’s protocols, reverse transcription and real-time PCR was conducted regularly using the kits (Toyobo, Osaka, Japan.), and the genes’ expression levels were detected with reference to the *GAPDH* gene. The primer sequences are listed in Additional file 1: Table S1.

### Western blot

Cartilage tissues induced by hMSCs were ground by a high-throughput tissue grinder after adding protease inhibitor cocktail and RIPA lysis buffer. Proteins were exacted and the concentration was quantified. The proteins were subjected to 6%-15% SDS-PAGE gel and were then transferred to PVDF transfer membranes. The bands were blocked and incubated with primary and secondary antibodies. Finally, they were visualized with the ECL kit and quantified by the ImageJ software. The dilutions of antibodies used in western blot are listed in Additional file 1: Table S2.

### Antibodies and reagents

Antibodies against aggrecan (ACAN), collagen type II (COL2A1), and SRY-box transcription factor 9 (SOX9) were purchased from Abcam. Antibodies against PI3K/AKT signaling protein (PI3K, p-PI3K, AKT, p-AKT, mTOR, and p-mTOR), GAPDH and secondary antibodies were purchased from Cell Signaling Technology *Inc.* Antibody against signal-induced proliferation-associated 1 like 2 (SIPA1L2) was purchased from Bioss. Human recombinant irisin was purchased from Novoprotein. 3MA (HY-19312) and GSK690693 (HY-10249) were purchased from MCE. The catalog numbers and company brands of antibodies used in this study are listed in Additional file 1: Table S3.

### Immunohistochemistry (IHC)

Cartilage tissues were fixed and embedded in paraffin. The sections were deparaffinized and immersed in 65 °C citrate buffer overnight for antigen retrieval. On the second day, the sections were incubated with 0.5% triton solution, 3% peroxidase, 5% bovine serum albumin, and anti-ACAN and anti-COL2A1 (1:100). On the next day, sections were incubated in secondary antibodies and were then detected. At last, images were obtained using an Olympus BX63 microscope. The dilutions of antibodies used in IHC are listed in Additional file 1: Table S2.

### Safranin O and Alcian Blue staining

After being fixed, embedded, and deparaffinized, the sections were treated with 0.5% Safranin O or Alcian Blue solutions. In the indicating time, sections were washed with water for 2 min. Finally, an Olympus BX63 microscope was used for photography at suitable magnifications.

### Immunofluorescence (IF)

Cartilage tissues were fixed, embedded, and deparaffinized. After being treated with 0.5% triton solution and 5% bovine serum albumin, the sections were incubated with primary antibodies at the concentration of 1:100. On the second day, they were incubated with Alexa Fluor®488-labeled or Alexa Fluor®594-conjugated secondary antibodies (1:100) for 1 h. Next, they were labeled with DAPI and photographed with an Olympus BX63 microscope. The dilutions of antibodies used in IF are listed in Additional file 1: Table S2.

### Plasmid transfection for SIPA1L2 over-expression

The oligonucleotides were packaged in plasmid vectors by GeneChem (Shanghai, China). When cells reached at 70–90% confluence, the over-expressed-SIPA1L2 (OE-SIPA1L2) and the (OE-NC) plasmid were added to the MSC-containing medium with additional Lipofectamine 3000 reagent at the concentration of approximately 2500 ng per well of the 6-well plate. After 6–8 h, the medium was replaced, and then the cells were cultured for following experiments.

### CCK-8 test

The hMSCs were plated in 96-well plates. After being cultured for 12 h, irisin at different concentrations was added in the medium. After 24 and 48 h, hMSCs were washed with PBS, and 10% CCK-8 reagent was added. Finally, we measured the absorbance by using the microplate reader at 450 nm.

### Statistical analysis

All statistical analyses were conducted using GraphPad Software 8.0, and SPSS 20.0 statistical software package. The results were presented as mean ± SD. Statistical analysis was performed using two-tailed independent Student’s t-test or one-way ANOVA, followed by Dunnett’s post hoc test. A *P* value of less than 0.05 was considered to indicate statistical significance.

## Results

### Irisin enhances chondrogenic differentiation of hMSCs

**Fig. 1:**
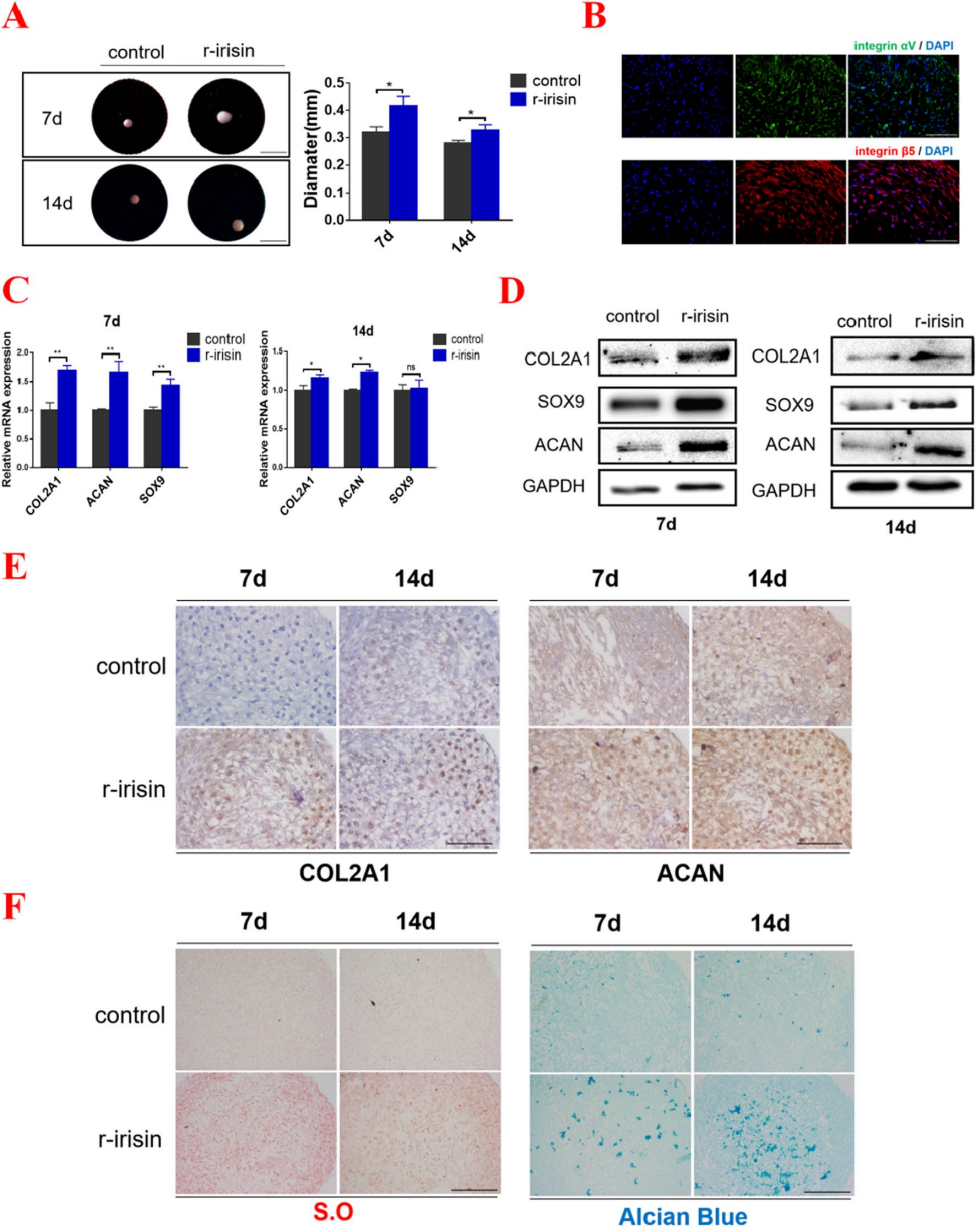
Irisin enhances chondrogenic differentiation of hMSCs. **A** Macro-images of induced-cartilage tissues were taken on Day-7 and Day-14 (Left panel). (Magnification: × 15, scale bar: 2 mm). The relative diameter of cartilage tissues in controls and r-irisin-treated group (Right panel). **B** The expression of integrin αVβ5 receptor in hMSCs-induced cartilage tissue. (Magnification: × 400, scale bar: 50 μm). **C** The mRNA expression levels of *COL2A1*, *ACAN* and *SOX9* were detected by qPCR in different groups on Day-7 and Day-14. **D** Protein expression levels of COL2A1, ACAN and SOX9 were detected by western blot in different groups on Day-7 and Day-14. **E** Immunohistochemistry for COL2A1 and ACAN on Day-7 and Day-14 in different groups. (Magnification: × 200, scale bar: 100 μm). **F** Safranin O and Alcian Blue staining for cartilage matrix metabolism on 7 and 14 days in different groups. (Magnification: × 200, scale bar: 100 μm). The levels were determined using the ImageJ software. The concentration of irisin used in this figure was 100 ng/ml. *P < 0.05, **P < 0.01 compared with the control-group

To determine the effects of irisin on chondrogenic differentiation of hMSCs, we firstly detected the proliferation effect of irisin on MSCs by CCK-8 test. As shown in Additional file 2: Fig. S1A-B, irisin at the concentration of 100 ng/ml showed no significant effect on MSCs within 24 and 48 h (Additional file 2: Fig. S1A-B). Next, irisin was added to chondrogenic medium at a concentration of 100 ng/ml. As shown in Fig. 1A, we found that the induced cartilage tissues treated with irisin were larger than controls on day 7 and 14, respectively (Fig. 1A). Meanwhile, we detected the expression of irisin receptor integrin αVβ5 in cartilage tissues using immunofluorescence (Fig. 1B). Subsequently, mRNA and proteins were extracted from induced-cartilage pellets, and results showed that irisin treatment significantly enhanced the expression levels of COL2A1, ACAN, and SOX9 (Fig. 1C, D and Additional file 2: Fig. S1C-D). In addition, the results from immunohistochemistry showed that irisin treatment could up-regulate the expression levels of cartilage markers (COL2A1 and ACAN) on day 7 and 14 (Fig. 1E and Additional file 2: Fig. S1E). Furthermore, Safranin O and Alcian Blue staining were used for evaluating the metabolism of cartilage matrix, and the results revealed that irisin treatment significantly enhanced cartilage matrix synthesis in the process of chondrogenic differentiation of hMSCs both on day 7 and 14 (Fig. 1F and Additional file 2: Fig. S1F). These outcomes showed that irisin enhanced the chondrogenic differentiation of hMSCs.

### Irisin activates the Rap1 and PI3K/AKT pathways

**Fig. 2:**
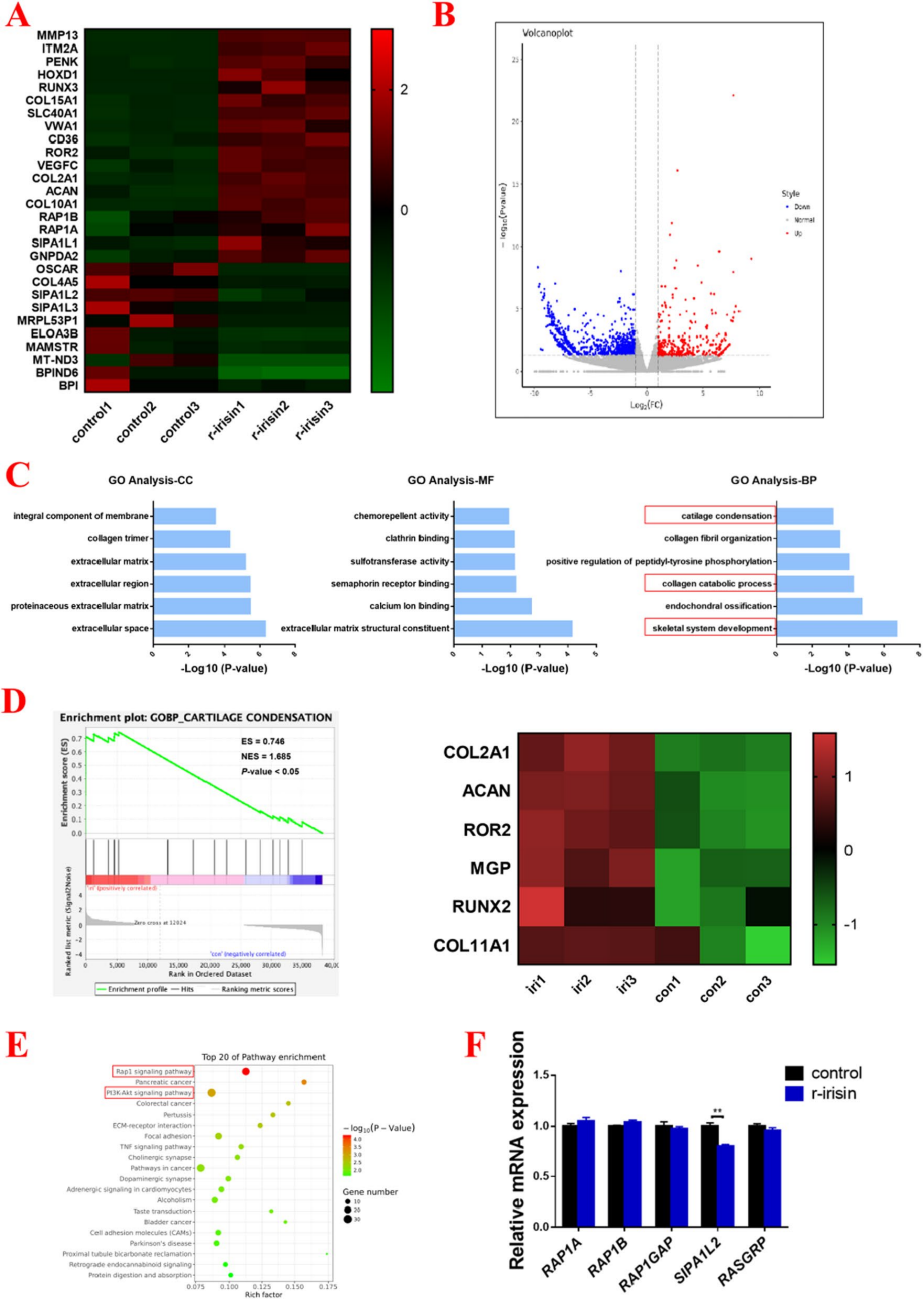
Irisin activates the Rap1 and PI3K/AKT pathways. **A** The heatmap of DEGs, as detected by RNA-seq in hMSCs induced-cartilage tissue treated with and without irisin for 7 d (n = 3 biological replicates). **B** The volcano map of genes distribution. **C** GO terms with the most significant *P* values. *BP: biological process, CC: cellular component, MF: molecular function.* **D** GSEA of positively associated cartilage condensation process (Left panel) and cartilage condensation hallmark genes treated with irisin compared with vehicle (Right panel). **E** The top 20 of pathways enrichment by KEGG analysis. **F** The mRNA expression levels of key genes (*RAP1A*, *RAP1B*, *RAP1GAP*, *SIPA1L2*, *RASGRP*) in Rap1 signaling pathway. The concentration of irisin used in this figure was 100 ng/ml. *P < 0.05, **P < 0.01 compared to control

To further explore the possible mechanisms accounting for the role of irisin on the chondrogenic differentiation of hMSCs, RNA sequencing was performed in hMSC-induced cartilage tissue between controls and irisin-treated groups on day 7. As shown in Fig. 2A, B, the differentially expressed genes were screened out, and notably, irisin treatment effectively up-regulated the expression level of genes known to participate in the process of cartilage condensation and development, including *COL2A1* and *ACAN* (Fig. 2A, B). Besides, the expression levels of fibrocartilage and cartilage hypertrophy associated genes (*COL10A1* and *MMP13*) were also up-regulated in the irisin treatment group (Fig. 2A and Additional file 2: Fig S2). Next, GO enrichment analysis was performed and revealed that skeletal system development, collagen catabolic process, and cartilage condensation were the biological processes which were affected by irisin treatment (Fig. 2C). Furthermore, gene-set enrichment analysis (GSEA) was performed and prompted that the cartilage condensation process was up-regulated in the irisin-treated group (ES = 0.746, NES = 1,685, and *P* < 0.05), and the relative genes (*COL2A1, ACAN, ROR2, MGP, RUNX2,* and *COL11A1*) were significantly up-regulated (Fig. 2D). Besides, KEGG pathway analysis identified that the Rap1 and PI3K/AKT signaling pathway were activated in the irisin-treated group (Fig. 2E). Verification of the special gene expressions in Rap1 signaling pathway was performed, and we found that the expression of *SIPA1L2* was substantially suppressed in the irisin-treated group (Fig. 2F).

### Irisin activates the Rap1 signaling pathway by suppressing the expression of SIPA1L2

**Fig. 3:**
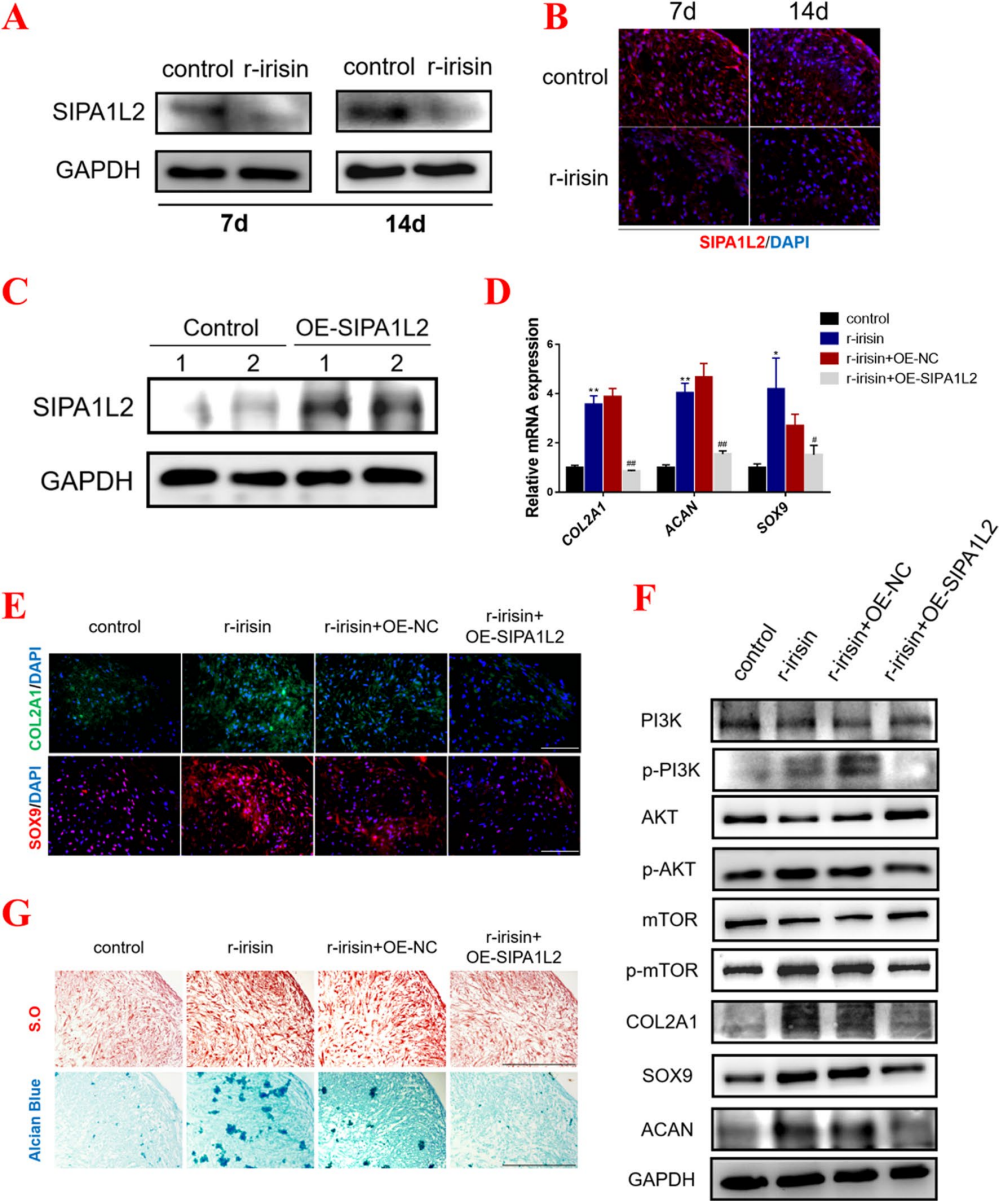
Irisin activates the Rap1 signaling pathway by suppressing the expression of SIPA1L2. **A** Protein expression levels of SIPA1L2 were detected by western blot in control and irisin-induced group on 7 and 14 days. **B** Immunofluorescence for SIPA1L2 on 7 and 14 days in different groups. (Magnification: × 400, scale bar: 50 μm). **C** Transfection efficiency verification of SIPA1L2 by western blot. **D** The mRNA expressions of *COL2A1*, *ACAN*, and *SOX9*. **E** Immunofluorescence of COL2A1 and SOX9 of MSCs-induced cartilage tissue on 7 days. (Magnification: × 400, scale bar: 50 μm). **F** Protein expression levels of PI3K, p-PI3K, AKT, p-AKT, mTOR, p-mTOR, COL2A1, ACAN and SOX9 were detected by western blot in different groups. **G** Safranin O and Alcian Blue staining in different groups. (Magnification: × 200, scale bar: 100 μm). The concentration of irisin used in this figure was 100 ng/ml. ^*^P < 0.05, ^**^P < 0.01 compared with the control group. ^#^*P* < 0.05, ^##^*P* < 0.01 compared with the irisin + OE-NC group

To investigate the function of Rap1 signaling pathway in the process of chondrogenic differentiation, we subsequently assessed the expression level of the key factor, SIPA1L2, a Rap1 GTPase activating protein, which was significantly inhibited with irisin treatment (Fig. 2A, F). The results from Western blot and immunofluorescence showed that the expression level of SIPA1L2 was evidently down-regulated with irisin treatment (Fig. 3A, B and Additional file 2: Fig. S3A-B). To explore the capability of SIPA1L2 in the process of chondrogenic differentiation, *SIPA1L2* was over-expressed using plasmid transfection, and the over-expression efficiency was tested using Western blot (Fig. 3C and Additional file 2: Fig. S3C). As shown in Additional file 2: Fig. S3D-E, we found that the induced-cartilage pellets treated with irisin were larger than controls, while this change was obviously inhibited in the OE-SIPA1L2 group (Additional file 2: Fig. S3D-E). Subsequently, we collected the hMSCs-induced cartilage tissue on day 7, and the results from qPCR, Western blot and immunofluorescence revealed that OE-SIPA1L2 significantly inhibited the expression of *COL2A1*, *ACAN*, and *SOX9*, while also supressed the activation of the PI3K/AKT/mTOR pathway (Fig. 3D–F). The results from Alcian Blue and Safranin O staining also showed that OE-SIPA1L2 could negatively regulate cartilage matrix synthesis in the irisin-treated group (Fig. 3G and Additional file 2: Fig. S3F). These findings indicated that the expression level of *SIPA1L2* was suppressed, and the Rap1 signaling pathway was activated with irisin treatment.

### Irisin enhances chondrogenic differentiation of hMSCs by activating the PI3K/AKT pathway

**Fig. 4:**
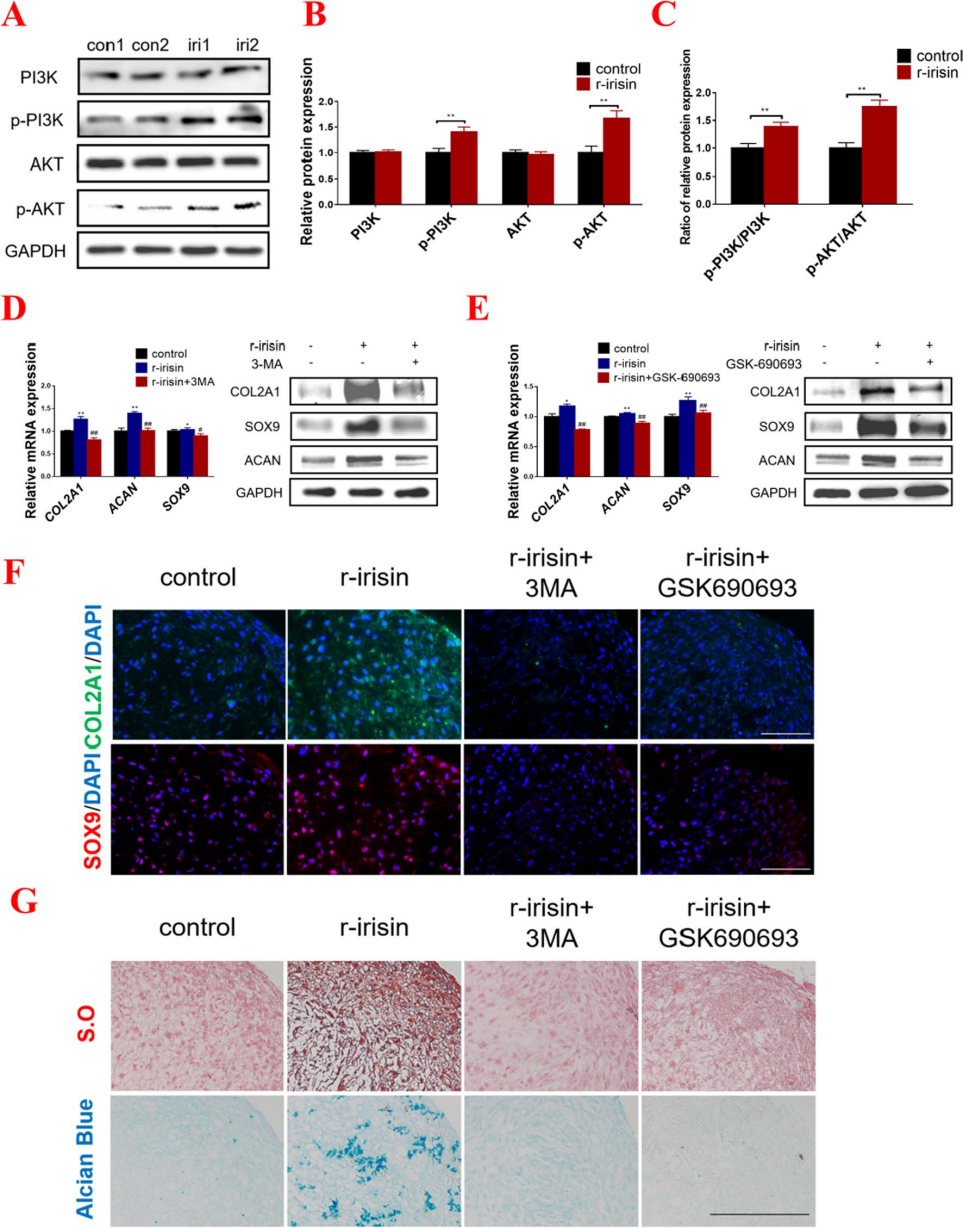
Irisin enhances chondrogenic differentiation of hMSCs by activating the PI3K/AKT pathway. **A** Protein expression levels of PI3K, p-PI3K, AKT and p-AKT were detected by western blot in controls and irisin-treated groups on 7 days. **B** Quantitative expression levels of PI3K, p-PI3K, AKT and p-AKT using Image J software. **C** The ratios of relative protein expression of p-PI3K to relative protein expression of PI3K (p-PI3K/PI3K), and p-AKT to relative protein expression of AKT (p-AKT/AKT). **D** The mRNA and protein expression levels of COL2A1, ACAN, and SOX9 were detected by qPCR and western blot in control, r-irisin, and r-irisin + 3MA-treated group. **E** The mRNA and protein expressions of COL2A1, ACAN, and SOX9 were detected by qPCR and western blot in control, irisin, and irisin + GSK690693-treated group. **F** Immunofluorescence of COL2A1 and SOX9 of hMSCs-induced cartilage tissues. (Magnification: × 400, scale bar: 50 μm). **G** Safranin O and Alcian Blue staining were performed after 7 days of differentiation in different group. (Magnification: × 200, scale bar: 100 μm). The concentration of irisin used in this figure was 100 ng/ml. *P < 0.05, **P < 0.01 compared with the control group. ^#^*P* < 0.05, ^##^*P* < 0.01 compared with the irisin-treated group

It has been demonstrated by previous studies, the PI3K/AKT signaling pathway, which was involved in the process of chondrogenic differentiation, was regulated by Rap1 signaling pathway [25, 26]. Meanwhile, in our study the activation of the PI3K/AKT signaling pathway was detected in irisin-treated group by KEGG analysis (Fig. 2E). Hence, the expression level of the key molecules of the PI3K/AKT pathway were detected. As shown in Fig. 4A–C, the phosphorylation levels of PI3K and AKT as well as the ratio of p-PI3K/PI3K and p-AKT/AKT were positively regulated with irisin treatment (Fig. 4A–C). To further explore the mechanism, two well-established small-molecule antagonists of PI3K/AKT pathway, 3-Methyladenine (3MA, a PI3K inhibitor) and GSK690693 (an ATP-competitive pan-AKT inhibitor) were employed. Compared with the control group, irisin treatment obviously enlarged the cartilage pellets, while 3MA and GSK690693 treatment decreased the diameter of cartilage tissues (Additional file 2: Fig. S4A). In addition, the detection of cartilage matrix components (COL2A1, ACAN, and SOX9) revealed that irisin promoted chondrogenic differentiation, while 3MA and GSK690693 inhibited these changes (Fig. 4D, E). And the results from immunofluorescence were consistent with the above-mentioned findings (Fig. 4F). Next, to evaluate the cartilage matrix synthesis and accumulation, Safranin O and Alcian Blue staining were used. Our data suggested that irisin treatment could promote the synthesis of cartilage matrix, while the effects were partially reversed by 3MA and GSK690693 (Fig. 4G and Additional file 2: Fig. S4B). These results demonstrated that the PI3K/AKT pathway was activated on the process of chondrogenic differentiation of irisin-induced hMSCs.

### Irisin-mediated up-regulation of miR-125b-5p targeting SIPA1L2 to promote chondrogenic differentiation of hMSCs

**Fig. 5:**
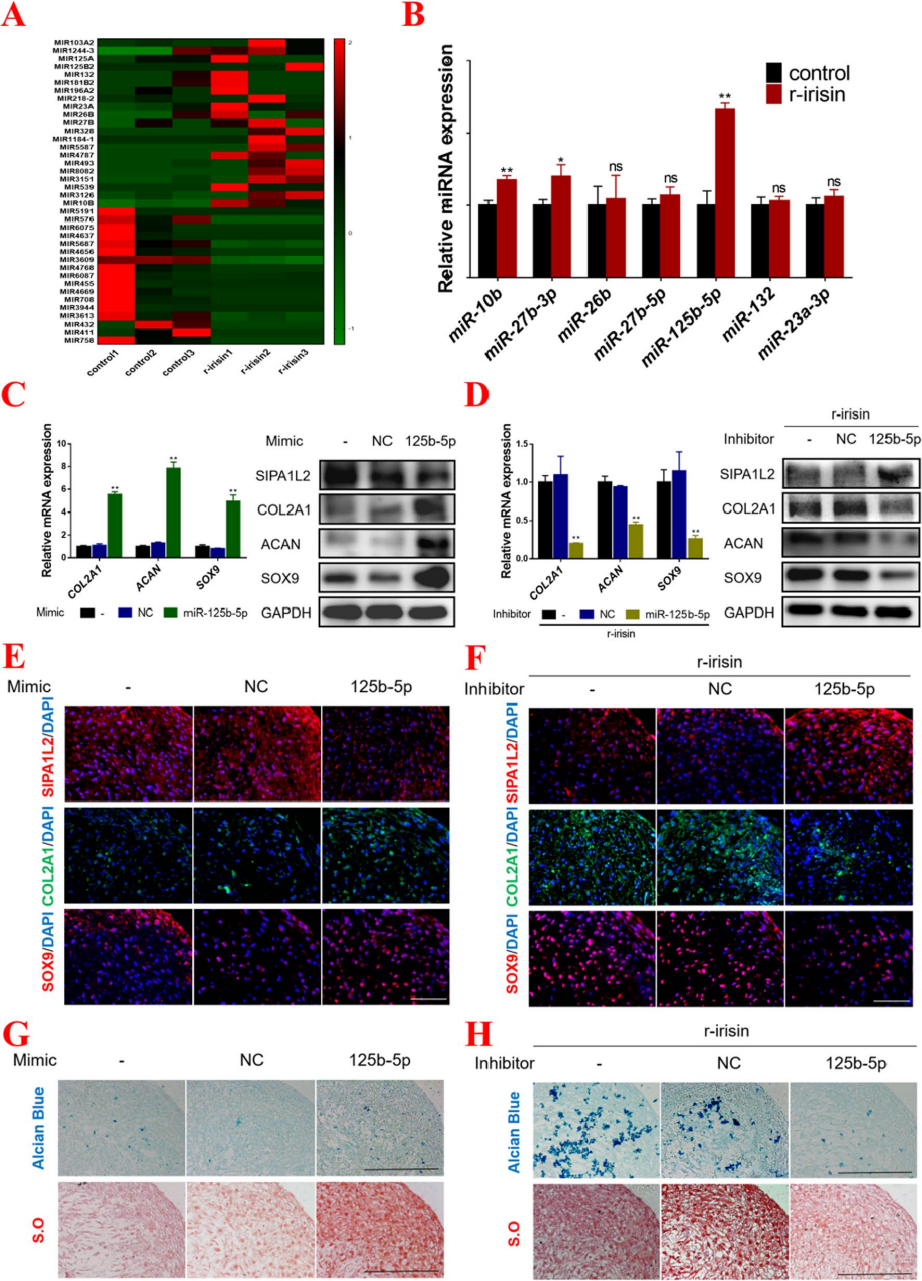
Irisin-mediated up-regulation of miR-125b-5p targeting SIPA1L2 to promote chondrogenic differentiation of hMSCs. **A** Human MSCs was seeded in 24-wells plates via high-density micromass culture, and chondrogenic differentiation was induced in chondrogenic medium with or without irisin (100 ng/ml) treatment for 7 days. Differently miRNA expressions in control and irisin-treated group were shown. **B** Relative expressions of the indicated miRNAs in cartilage tissues detected by qPCR. **C** The mRNA expressions (Left panel) of *COL2A1*, *ACAN*, and *SOX9*, and the protein expressions (Right panel) of SIPA1L2, COL2A1, ACAN, and SOX9 in the control, *NC*, and *miR-125b-5p* mimics transduction groups. **D** The mRNA expressions (Left panel) of *COL2A1*, *ACAN*, and *SOX9*, and the protein expressions (Right panel) of SIPA1L2, COL2A1, ACAN, and SOX9 in the control, *NC*, and *miR-125b-5p* inhibitor transduction groups under irisin treatment. **E**, **F** The expressions of SIPA1L2, COL2A1, and SOX9 were detected by immunofluorescence combined with DAPI staining for the nuclei in different groups. (Magnification: × 400, scale bar: 50 μm). **G**, **H** Safranin O and Alcian Blue staining were performed in cartilage tissue paraffin sections with different treatments as shown. (Magnification: × 200, scale bar: 100 μm). The concentration of irisin used in this figure was 100 ng/ml. *P < 0.05, **P < 0.01 compared with the control group or NC group

It has been found that miRNAs were involved in regulating the chondrogenic differentiation of hMSCs [22, 27]. We compared miRNA expression in controls and irisin-treated cartilage tissue (Fig. 5A), and we selected 12 genes among these differentially over-expressed miRNAs-targeting *SIPA1L2* gene from the database, and then we detected the expression levels of these miRNAs, and the results from qPCR showed that *miR-125b-5p* was most significantly up-regulated with irisin treatment (Fig. 5B). Thus, we transduced hMSCs with *miR-125b-5p* mimics and irisin-treated hMSCs with *miR-125b-5p* inhibitor. As expected, the results showed that the expression levels of chondrogenic markers (*COL2A1*, *ACAN,* and *SOX9*) were up-regulated with miR-125b-5p mimics transduction, while being down-regulated in irisin-treated hMSCs with miR-125b-5p inhibitor transduction (Fig. 5C, D). Consistently, the results from immunofluorescence showed that *miR-125b-5p* mimics inhibited the level of SIPA1L2 and increased the expressions of typical chondrogenic markers COL2A1 and SOX9. Correspondingly, the transduction of hMSCs with *miR-125b-5p* inhibitor partly reversed the expression level of SIPA1L2 under irisin treatment and abrogated the positive effects of irisin on COL2A1 and SOX9 expression levels (Fig. 5E, F). Moreover, Alcian Blue and Safranin O staining showed more abundant cartilage matrix synthesis in the *miR-125b-5p* mimics transduction group compared with the controls (Fig. 5G and Additional file 2: Fig. S5A). On the contrary, the promotional effect of irisin on chondrogenic differentiation was abrogated by transducting *miR-125b-5p* inhibitor (Fig. 5H and Additional file 2: Fig. S5B). These data suggested that irisin treatment could up-regulate *miR-125b-5p* targeting *SIPA1L2*, enhancing the chondrogenic differentiation of hMSCs.

## Discussion

Irisin, known as a polypeptide fragment of FNDC5, produced in abundance by skeletal muscle, is a hormone-like myokine that leads to increased energy expenditure as well as regulation of glucose homeostasis and bone metabolism [22, 27]. Ample evidence is emerging in support of irisin’s physiological relevance in development of the skeletal system and chondroprotective actions [20, 21, 28]. Moreover, with a wide spectrum for the repair of defective or damaged cartilage tissue, MSCs are currently undergoing trials for articular cartilage repair in the field of tissue engineering [8, 29–31]. Nevertheless, the effects and potential mechanisms of irisin on chondrogenic differentiation of hMSCs are elusive yet.

**Fig. 6:**
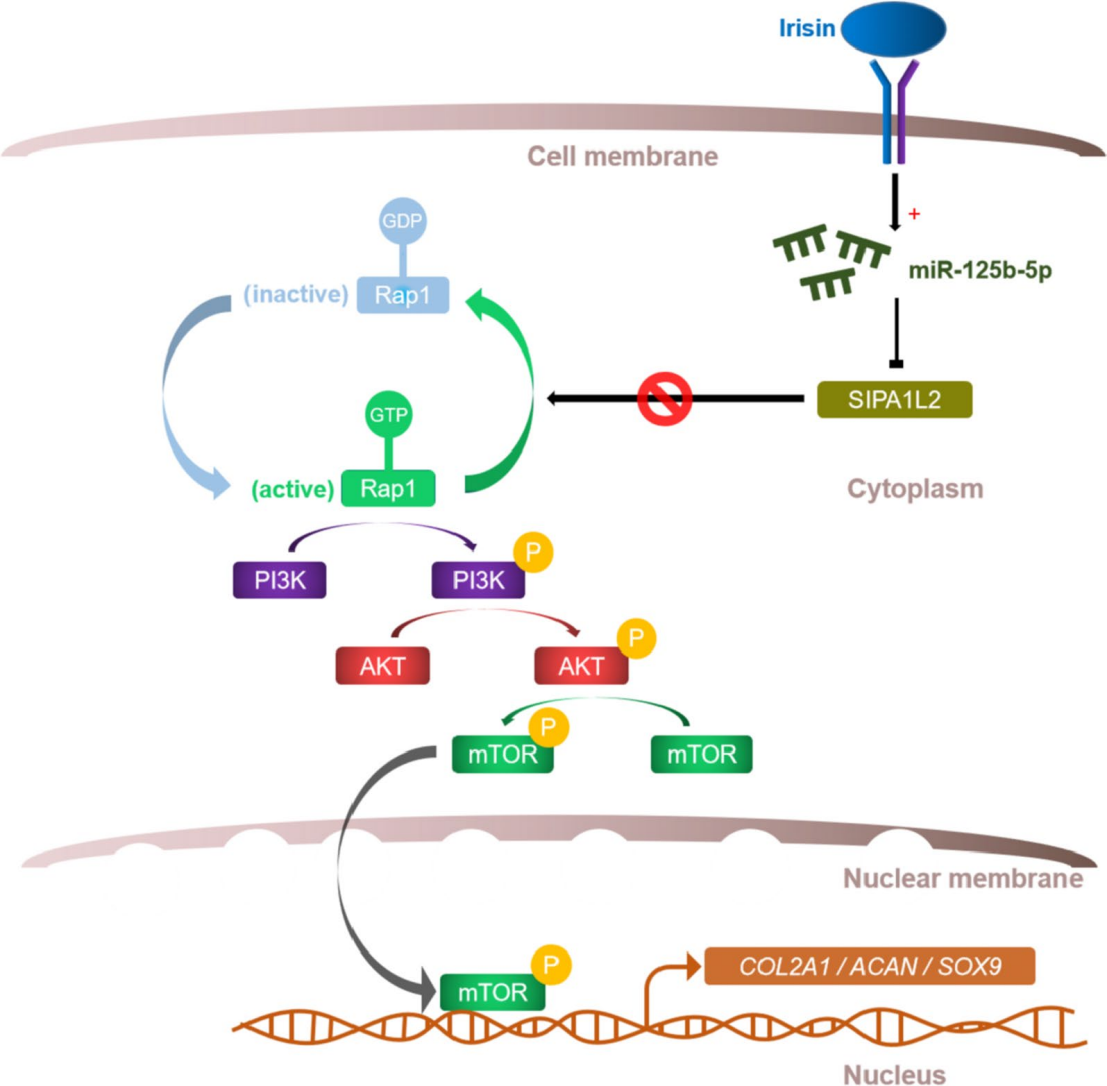
Schematic diagram highlighting the primary findings of this study. Irisin up-regulates *miR-125b-5p,* targeting *SIPA1L2* and activates the Rap1/PI3K/AKT signaling axis, and consequently promoted the expression levels of chondrogenic differentiation genes (*COL2A1*, *ACAN*, and *SOX9*), enhancing chondrogenic differentiation in hMSCs via integrin receptor.

In the study, we found that irisin played a promoting role on chondrogenic differentiation of hMSCs. Mechanism-wise, we demonstrated that irisin activated the Rap1 signaling pathway and PI3K/AKT pathway. Further research revealed that irisin increased the level of *miR-125b-5p*, targeting *SIPA1L2*, which regulated the Rap1/PI3K/AKT axis as a Rap1 GTPase activating proteins and finally up-regulated the expression levels of chondrogenic differentiation genes *COL2A1*, *ACAN*, and *SOX9* (Fig. 6). Our research evidenced the positive effects of irisin on chondrogenic differentiation of hMSCs, and detected its molecular mechanism that may lead to new ideas for the application of hMSCs implantation in the field of tissue engineering to repair articular cartilage lesions.

The process of endochondral osteogenesis mainly includes mesenchymal condensation, chondrocyte differentiation, chondrocyte maturation, cartilage vascularization and bone collar formation [32, 33]. Nowadays cartilage tissue regeneration and engineering are considered as two primary treatment methods for cartilage injuries [34], while MSCs were considered as the most appropriate cell source for cartilage tissue engineering due to the ability of chondrogenic differentiation [35]. Meanwhile, a hurdle to using MSCs in a cartilage application is creating a chondrocyte that will not enter hypertrophy and endochondral ossification. In the present study, we found that irisin treatment could enhance chondrogenic differentiation of hMSC, enlarge the cartilage pellets on day 7 and 14 of chondrogenic differentiation, and up-regulate the expression levels of cartilage-associated markers (COL2A1 and ACAN) at both mRNA and protein levels. On the other hand, the results from RNA-seq indicated that the bone-associated proteins like clathrin, calcium binding and endochondral ossification-associated genes were affected by irisin treatment, suggesting that irisin treatment might be also involved in the later period of endochondral osteogenesis, and further studies to confirm the effects and clarify the mechanism are needed. Thus, we think it is still a huge challenge that using irisin with hMSCs in the field of articular cartilage injuries, and further studies are warranted.

Rap1 protein, as a member of the Ras superfamily of small GTPases, is regulated by both Rap1 GTPase activating proteins and Rap1-specific guanine nucleotide exchange factors, which can activate the following ERK signaling, PI3K/AKT signaling and other downstream pathways [36–38]. As a Rap1 GTPase activating protein, SIPA1L2 promotes the intrinsic GTPase activity of Rap1 that catalyzes the hydrolysis of GTP to GDP, and consequently, inhibits the downstream pathways including ERK signaling and PI3K/AKT signaling [39, 40]. According to available evidence, the PI3K/AKT signaling pathway was essential for normal cartilage development, metabolism, and degradation [25, 26, 41, 42]. Nevertheless, the molecular mechanisms governing Rap1/PI3K/AKT on the regulation of chondrogenic differentiation are still unclear. Our results suggested that irisin treatment effectively down-regulated the expression of SIPA1L2, helped to maintain activation of long-term Rap1 protein, and activated the downstream PI3K/AKT pathway in the process of induced-chondrogenic differentiation of hMSCs.

MiRNAs are short, noncoding, endogenous oligonucleotides which can be found in almost all cells. Through post-transcriptional gene silencing, they can regulate gene expression [27, 43]. Accumulated evidences have shown that post-transcriptional regulation by microRNAs (miRNAs) was involved in the osteogenic and chondrogenic differentiation of MSCs [22, 44]. It was a practical approach to mediate the chondrogenic differentiation of MSCs by manipulating the expression of specific miRNAs [22, 45, 46]. A variety of miRNAs participated in the processes of irisin on osteogenic differentiation of MSCs, browning of white adipocytes, and anti-inflammation [19, 47, 48]. Nevertheless, whether miRNAs participate in the process of irisin-enhanced chondrogenic differentiation of hMSCs is still unknown. Our study illustrated that irisin elevated chondrogenic differentiation in hMSCs through up-regulation of *miR-125b-5p* targeting *SIPA1L2*. Correspondingly, the administration of *miR-125b-5p* mimics successfully enhanced the chondrogenic differentiation. Meanwhile, the transduction of *miR-125b-5p* inhibitor practically abrogated the regulative effects of irisin on hMSCs’ chondrogenic differentiation. Thus, we hypothesize that it could be an effective therapy to modify hMSCs with miRNAs for diseases involving cartilage injury in the future.

## Conclusions

Collectively, we described that irisin effectively enhanced chondrogenic differentiation of hMSCs. Mechanistically, it was identified that irisin may activate the Rap1/PI3K/AKT signaling axis by up-regulating *miR-125b-5p* targeting *SIPA1L2*. In addition, the transductions of *miR-125b-5p* mimics or inhibitors can simulate or reverse the effects, respectively. More studies are warranted in the future to explore the application of irisin on tissue engineering and the clinical translation on cartilage regeneration.

## Supplementary Information

**Additional file 1**. Supplementary tables in this study.

**Additional file 2**. Supplementary figures and figure legends in this study.

## Data Availability

The data used to support the findings of this study are available from the corresponding authors upon request. The raw data of transcriptome sequencing had been submitted to the GEO (GSE201594). The Primers’ sequences for the real-time PCR are listed in Additional file 1: Table S1. The dilution, catalog numbers and company brands of antibodies used in this study are listed in Additional file 1: Tables S2 and S3.

## Abbreviations

hMSCs: Human mesenchymal stem cells
FNDC5: Fibronectin component including protein 5
ACAN: Aggrecan
COL2A1: Collagen type II
SOX9: SRY-box transcription factor 9
SIPA1L2: Signal-induced proliferation-associated 1 like 2
