# Supplementary material for: Irisin enhances chondrogenic differentiation of human mesenchymal stem cells via Rap1/PI3K/AKT axis
Source: Stem Cell Res Ther. 2022 Aug 3;13:392. doi: 10.1186/s13287-022-03092-8 (PMC9351134; doi:10.1186/s13287-022-03092-8)
Supplement: Supplementary file 1 — Additional file 1. Supplementary tables in this study. [file 13287_2022_3092_MOESM1_ESM.docx]

**Supplementary TABLEs**

**Irisin enhances chondrogenic differentiation of human mesenchymal stem cells via Rap1/PI3K/AKT axis.**

**Table S1. Primers’ sequences for the real-time PCR.**

| Gene primer names | Primer sequence(5’-3’) |
| --- | --- |
| *GAPDH*-F  *GAPDH*-R  *COL2A1*-F  *COL2A1*-R  *ACAN*-F  *ACAN*-R  *SOX9*-F  *SOX9*-R  *RAP1A*-F  *RAP1A*-R  *RAP1B*-F  *RAP1B*-R  *RAP1GAP*-F  *RAP1GAP*-R  *RASGRP2*-F  *RASGRP2*-R  *SIPA1L2*-F  *SIPA1L2*-R  *miR-26b*-F  *miR-26b*-R  *miR-27b-3p*-F  *miR-27b-3p*-R  *miR-27b-5p*-F  *miR-27b-5p*-R  *miR-10b*-F  *miR-10b*-R  *miR-125b-5p*-F  *miR-125b-5p*-R  *miR-132*-F  *miR-132*-R  *miR-23a-3p*-F  *miR-23a-3p*-R | AGAAAAACCTGCCAAATATGATGAC  TGGGTGTCGCTGTTGAAGTC  GGCAATAGCAGGTTCACGTACA  GAACATCGACCAACTCTACTCCG  ACTCTGGGTTTTCGTGACTCT  ACACTCAGCGAGTTGTCATGG  AGCGAACGCACATCAAGAC  GCTGTAGTGTGGGAGGTTGAA  CGTGAGTACAAGCTAGTGGTCC  CCAGGATTTCGAGCATACACTG  AGCAAGACAATGGAACAACTGT  TGCCGCACTAGGTCATAAAAG  GAGGAGGACTACATTCCATACCC  GCTGGTGATTTCGTGGTTGG  ACAATCCCGGAAGGACAACTC  GTCTATGTCGATTAGGCTGCTG  GACCCAAGGCAGTCACAAGAA  TAGGAGGCCATTCAGACACCC  CCGGGACCCAGTTCAAGTAA  CCCCGAGCCAAGTAATGGAG  GCTCTAGATTGCCAGGGATTACCACGCAA  CGGGATCCCTAGCATTCCCAGCAGGAGACAG  GACCTAGCACCATTTGAAATCA  GTCCAGGTTTTTTTTTTTTTTTTA  TACCCTGTAGAACCGAATTTGT  GTGCAGGGTCCGAGGT  GGGTCCCTGAGACCCTAAC  CAGTGCGTGTCGTGGAGT  TGGATCCCCCCCAGTCCCCGTCCCTCAG  TGAATTCGGATACCTTGGCCGGGAGGAC  GCGATCACATTGCCAGGG  AGTGCAGGGTCCGAGGTATT |

**Table S2. Dilution of antibodies used in this study.**

| Antibodies | Dilution |
| --- | --- |
| GAPDH  COL2A1  ACAN  SOX9  SIPA1L2  PI3K  p-PI3K  AKT  p-AKT  mTOR  p-mTOR  Anti-Rabbit IgG secondary antibodies  Anti-Mouse IgG secondary antibodies | 1:2000  WB (1:1000); IHC/IF (1:100)  WB (1:1000); IHC /IF(1:100)  WB (1:1000); IF(1:100)  WB (1:1000); IF(1:100)  WB (1:1000)  WB (1:1000)  WB (1:1000)  WB (1:1000)  WB (1:1000)  WB (1:1000)  WB (1:3000)  WB (1:3000) |

**Table S3. Catalog numbers and company brands of antibodies used in this study.**

| Antibodies | Company, Cat# |
| --- | --- |
| GAPDH  COL2A1  ACAN  SOX9  SIPA1L2  PI3K  p-PI3K  AKT  p-AKT  mTOR  p-mTOR  Anti-Rabbit IgG secondary antibodies  Anti-Mouse IgG secondary antibodies | Cell Signaling Technology Inc. Cat#5174  Abcam Inc. Cat# ab34712 and ab188570  Abcam Inc. Cat# ab3778  Abcam Inc. Cat# ab185966 and ab185230  Bioss Inc. Cat# bs-7927R  Cell Signaling Technology Inc. Cat#4255  Cell Signaling Technology Inc. Cat#17366  Cell Signaling Technology Inc. Cat#4691  Cell Signaling Technology Inc. Cat#4060  Cell Signaling Technology Inc. Cat#2983  Cell Signaling Technology Inc. Cat#5536  Cell Signaling Technology Inc. Cat#7074  Cell Signaling Technology Inc. Cat#7076 |
