# Supplementary material for: Irisin enhances chondrogenic differentiation of human mesenchymal stem cells via Rap1/PI3K/AKT axis
Source: Stem Cell Res Ther. 2022 Aug 3;13:392. doi: 10.1186/s13287-022-03092-8 (PMC9351134; doi:10.1186/s13287-022-03092-8)
Supplement: Supplementary file 2 — Additional file 2. Supplementary figures and figure legends in this study. [file 13287_2022_3092_MOESM2_ESM.docx]

**Supplement Figures and figure legends**

**Irisin enhances chondrogenic differentiation of human mesenchymal stem cells via Rap1/PI3K/AKT axis.**

**Fig. S1**

**
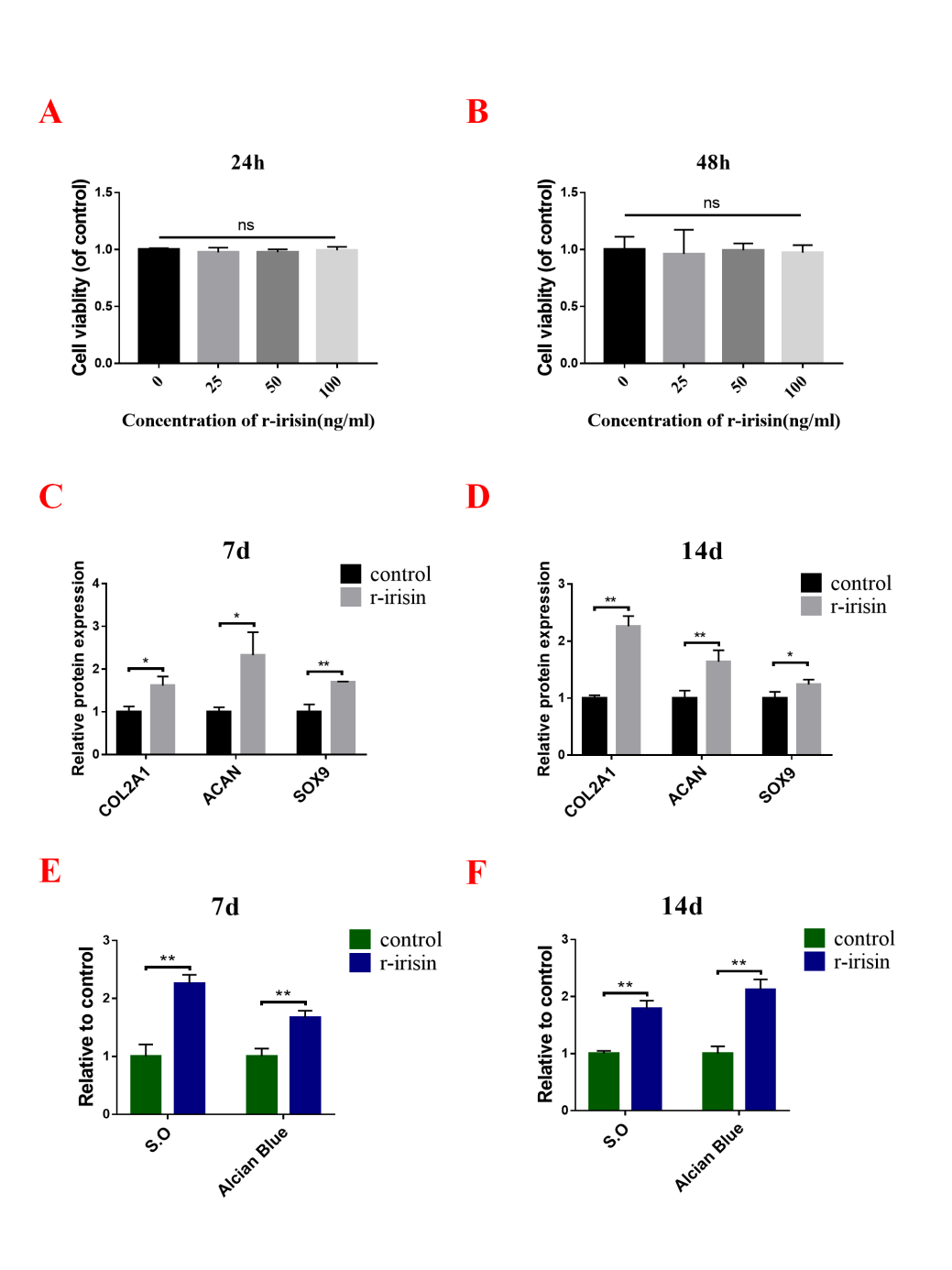
**

***Fig.*** ***S1*** ***Irisin enhances chondrogenic differentiation of hMSCs.*** (A-B) Dectection of irisin’s proliferation effect on MSCs at various concentrations at 24 and 48 h post-treatment using a CCK-8 assay (0, 25, 50, 100 ng/ml). (C-D) Quantitative expression levels of COL2A1, ACAN, and SOX9 on Day 7 and 14. (E-F) Quantitative data of Safranin O and Alcian Blue staining on Day 7 and 14. ^*^*P* < 0.05, ^**^*P* < 0.01 compared with the control group.

**Fig. S2**

**Fig. S2** The expression levels of fibrocartilage and cartilage hypertrophy associated genes (*COL10A1*, and *MMP13*) were detected by qPCR. ^**^*P* < 0.01 compared with the control group.

**Fig. S3**

**
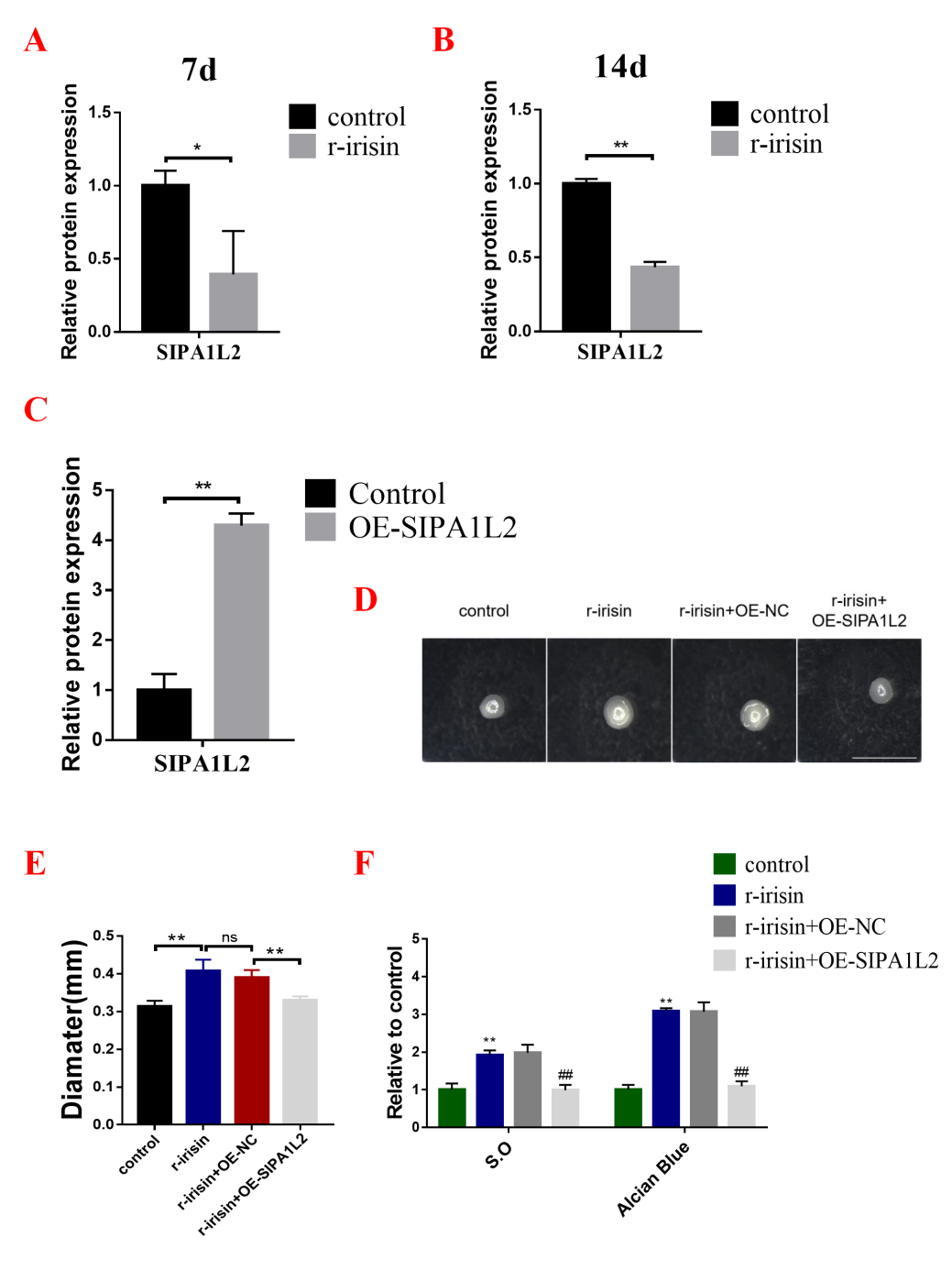
**

***Fig.*** ***S3*** ***Irisin activates the Rap1 signaling pathway by suppressing the expression of SIPA1L2.*** (A-B) Quantitative expressions levels of SIPA1L2 between controls and irisin-treated group on Day 7 and 14. (C) Quantitative expression levels of SIPA1L2 between controls and OE-SIPA1L2 group. (D) Macro-images of induced-cartilage tissues were taken on Day 7 in different groups. (Magnification: ×15, scale bar: 2mm). (E) The diameter of cartilage tissues in different group using Image J software. (F) Quantitative data of Safranin O and Alcian Blue staining in different groups. ^*^*P* < 0.05, ^**^*P* < 0.01 compared with the control group. ^#^*P* < 0.05, ^##^*P* < 0.01 compared with the r-irisin+OE-NC group.

**Fig. S4**

**
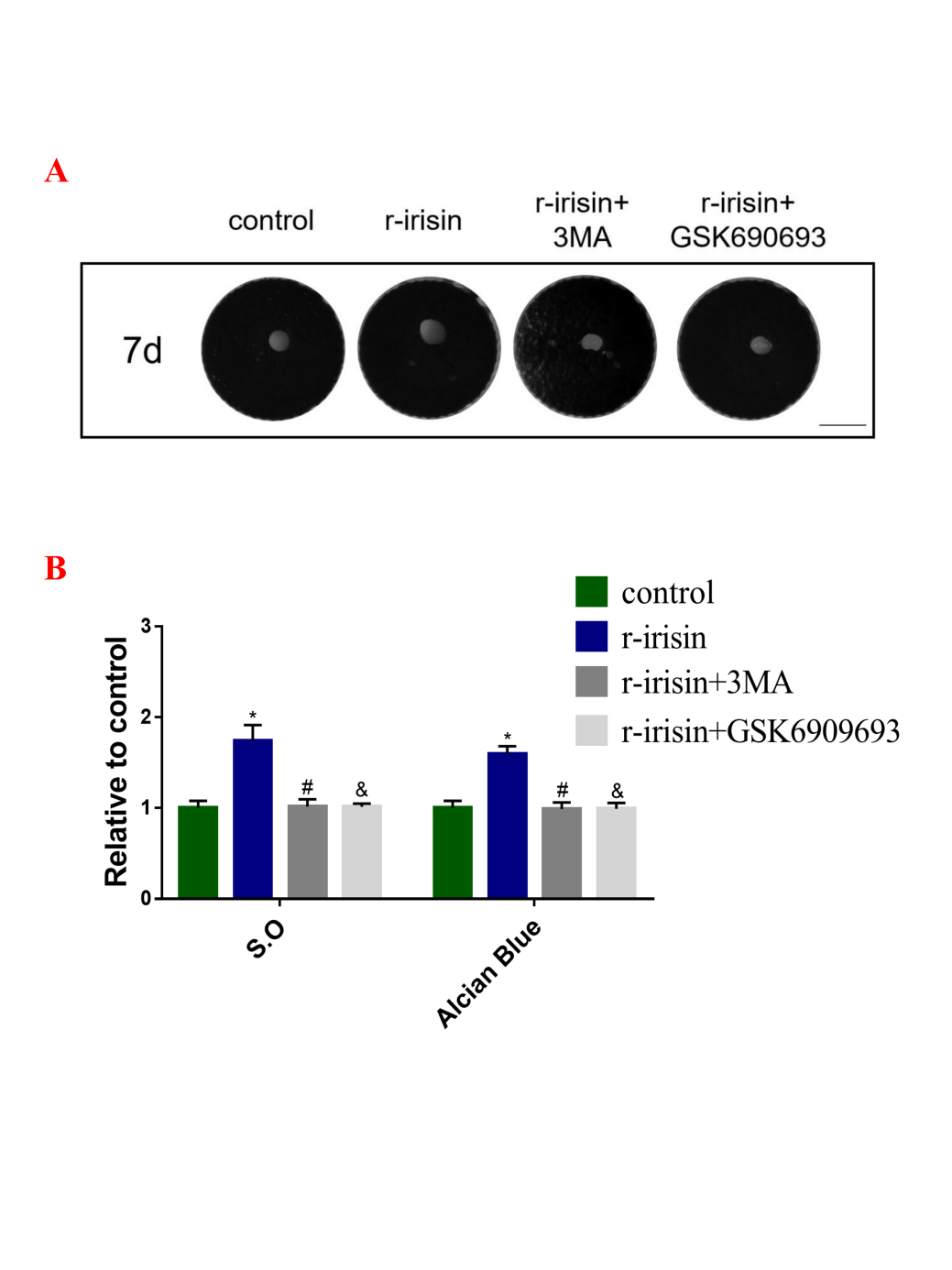
**

***Fig.*** ***S4*** ***Irisin enhances chondrogenic differentiation of hMSCs by activating the PI3K/AKT pathway.*** (A) Macro-images of induced-cartilage tissues were taken on Day 7 in different groups. (Magnification: ×15, scale bar: 2mm). (B) Quantitative data of Safranin O and Alcian Blue staining in different groups. ^*^*P* < 0.05, ^**^*P* < 0.01 compared with the control-group. ^#^*P* < 0.05, ^##^*P* < 0.01 compared with the r-irisin group. ^&^*P* < 0.05, ^&&^*P* < 0.01 compared with the r-irisin group.

**Fig. S5**


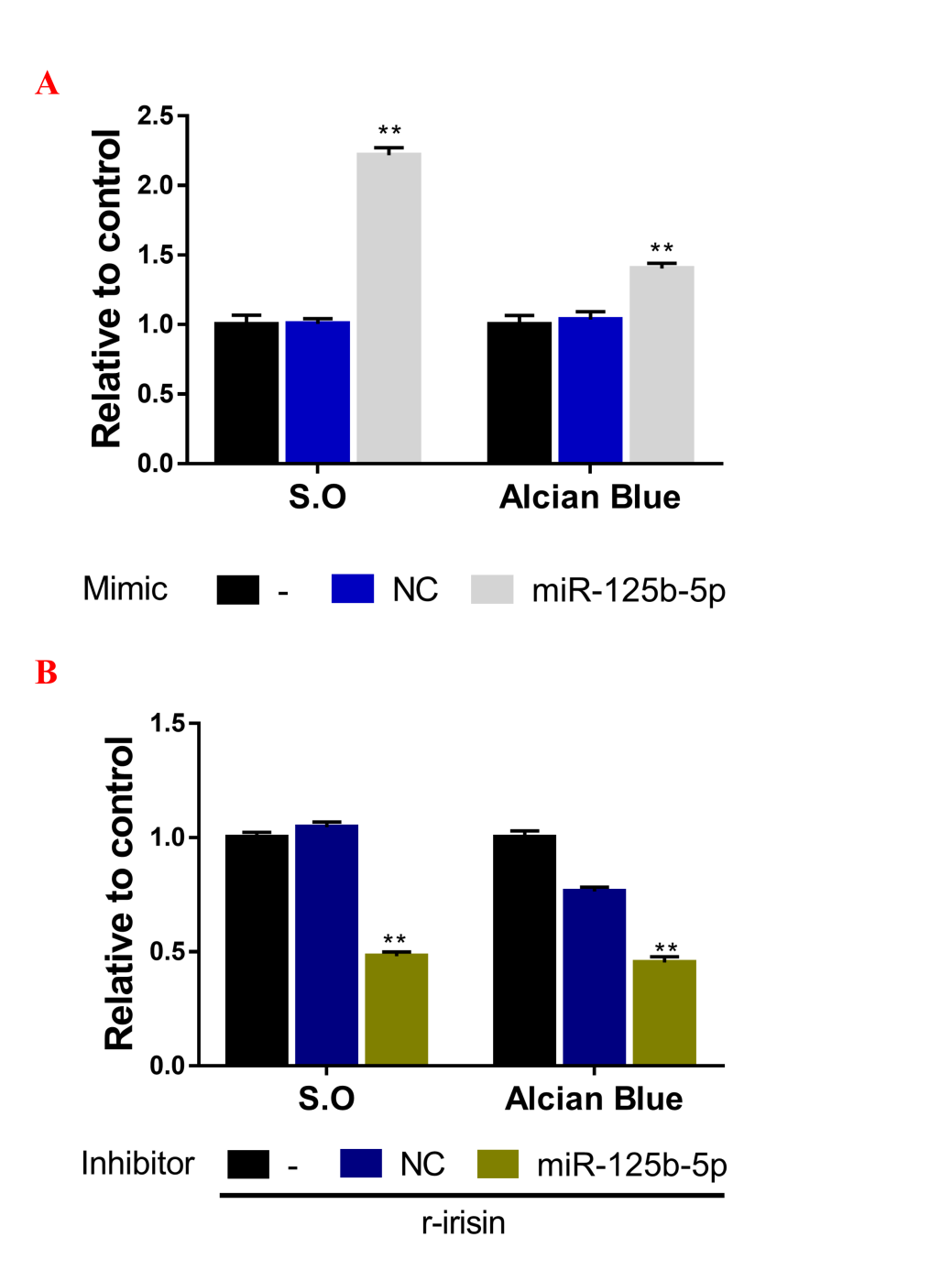


***Fig.*** ***S5*** ***Irisin mediated up-regulation of miR-125b-5p targeting SIPA1L2 to promote chondrogenic differentiation of hMSCs.*** (A-B) Quantitative data of Safranin O and Alcian Blue staining in different groups. ^*^*P* < 0.05, ^**^*P* < 0.01 compared with the control or NC group.
